# Whole Brain Size and General Mental Ability: A Review

**DOI:** 10.1080/00207450802325843

**Published:** 2009-05-19

**Authors:** J. Philippe Rushton, C. Davison Ankney

**Affiliations:** Departments of Psychology and Biology, University of Western Ontario, London, Ontario, Canada

**Keywords:** brain size, brain volume, intelligence, IQ scores, magnetic resonance imaging

## Abstract

We review the literature on the relation between whole brain size and general mental ability (GMA) both within and between species. Among humans, in 28 samples using brain imaging techniques, the mean brain size/GMA correlation is 0.40 (*N* = 1,389; *p* < 10^−10^); in 59 samples using external head size measures it is 0.20 (*N* = 63,405; *p* < 10^−10^). In 6 samples using the method of correlated vectors to distill *g*, the general factor of mental ability, the mean *r* is 0.63. We also describe the brain size/GMA correlations with age, socioeconomic position, sex, and ancestral population groups, which also provide information about brain–behavior relationships. Finally, we examine brain size and mental ability from an evolutionary and behavior genetic perspective.

Replication is a crucial part of the scientific process. Conceptual replication—across procedures, populations, and species—provides evidence of a reliable relationship—especially in the behavioral sciences that depend on correlational studies. This is our fifth review of the relation between brain size and general mental ability (GMA; Rushton & Ankney, 1995, 1996, 1997, 2007). It covers important new findings but also repeats some material that is still not widelyknown.

## THE BRAIN SIZE/GMA RELATIONSHIP

### Introduction

Throughout the nineteenth and early twentieth centuries, the relation between whole brain size and GMA was almost universally accepted (Broca, 1861; Darwin, 1871; Morton, 1849; Topinard, 1878). The renowned French neurologist Paul Broca (1824–1880) measured external and internal skull dimensions and weighed wet brains at autopsy and observed that mature adults averaged a larger brain than either children or the very elderly, skilled workers averaged a larger brain than the unskilled, and eminent individuals averaged a larger brain than the less eminent. Charles Darwin (1871) cited Broca's studies in *The Descent of Man* to support his theory of evolution:

No one, I presume, doubts that the large size of the brain in man, relatively to his body, in comparison with that of the gorilla or orang, is closely connected with his higher mental powers. We meet the closely analogous facts with insects, in which the cerebral ganglia are of extraordinary dimensions in ants; these ganglia in all the Hymenoptera being many times larger than in the less intelligent orders, such as beetles…

The belief that there exists in man some close relation between the size of the brain and the development of the intellectual faculties is supported by the comparison of the skulls of savage and civilized races, of ancient and modern people, and by analogy of the whole vertebrate series.

Darwin's cousin, Sir Francis Galton (1888), was the first to quantify the relation between brain size and GMA in living people. He multiplied head length by breadth by height and plotted the results against class of degree in more than 1,000 male undergraduates at Cambridge University. He reported that men who obtained high honors degrees had a brain size 2%–5% greater than those who did not. Years later, Karl Pearson (1906) reanalyzed Galton's data using the correlation coefficient he had invented for this type of analysis; he found *r* = 0.11.

Galton (1869) was also the first to formally propose the scientific concept of GMA. Subsequently, Spearman (1904, 1927) found the various GMA items and subtests correlated positively, indicating they all measured, in different degrees, one underlying dimension, which he dubbed “*g*,” the general factor of intelligence. Thus, a “spatial” test may be relatively high on *g* (mental rotation) or low (perceptual speed), a “verbal” test may be relatively high (reasoning) or low (fluency), as might a “memory” test be high (repeating a series in reverse order) or low (repeating a series in presented order). Scores on the *g* factor give the highest correlations with school grades, job performance, and other criteria such as 0.70–0.80 with academic achievement, 0.70 with military training, 0.30–0.60 with work performance, 0.30–0.40 with income, and 0.20 with conscientiousness, law abidingness, and longevity (Jensen, 1998; Lubinski, 2004).

Following World War II (1939–1945) and the revulsion evoked by Hitler's racial policies, craniometry became associated with extreme forms of racial prejudice. Research on brain size and intelligence virtually ceased, and the literature underwent vigorous critiques (Gould, 1978, 1981; Kamin, 1974; Tobias, 1970). However, as we shall show, modern studies confirm many of the earliest observations.

Anthropologist Van Valen (1974) rekindled discussion of brain size/GMA relations in *Homo sapiens* by reviewing a handful of studies using external head size which, when corrected for attenuation of measurement, gave an estimate of *r* = 0.30. He pointed out, it was predictable that correlations between IQ and overall brain size would be modest. First, much of the brain is not involved in producing GMA; thus, variation in size or mass of that tissue will lower the correlation. Second, the measures of GMA are imperfect.

In the appendix, we update our previous reviews of the human brain size/GMA literature (see also Gignac, Vernon, & Wickett, 2003; McDaniel, 2005). To be included, the samples had to be nonclinical; the published reference had to report an actual correlation; and the studies were not to be based on personal communications, unpublished papers, or works merely cited. We report the average or most representative correlation from those studies providing multiple correlations. Double entries were eliminated, particularly those emanating from the U.S. National Collaborative Perinatal Project (Broman et al., 1975, 1987). When possible, data were coded separately by sex. Corrections for body size typically were not included because many studies did not report this statistic, although age effects often were controlled for.

Appendix 1 shows the results of 28 studies that used brain imaging techniques such as magnetic resonance imaging (MRI) and computed tomography (CT) in a total of 1,389 normal subjects. The correlations with GMA range from 0.04 to 0.69, with an unweighted mean of 0.40 (when weighted by sample size, 0.38). Appendix 2 shows the results of 59 studies that recorded external head measurements in a total of 63,405 children, adolescents, and adults. The correlations range from 0.02 to 0.55 with an unweighted mean of 0.21 (when weighted by sample size, 0.20). We obtained the exact *p* values of all correlations in both Appendix 1 and Appendix 2 using Fisher's (1970, pp. 99–101) method for combining independent probabilities, and calculated the overall *p* values, which are less than 10^−10^ in both cases.

Six studies that used Jensen's (1998) *method of correlated vectors* to distill *g* from the subtests of an IQ test found the correlation with brain size is even higher (*r* = 0.63). The procedure consists of calculating the association between a column of quantified elements (such as *g* loadings) and any parallel column of independently derived scores (such as the correlation between GMA subtests and brain size). For example, Jensen (1994) found a simple correlation of 0.19 between head circumference and *g* on 17 cognitive tests among 286 adolescents became 0.64 using the method of correlated vectors. Wickett, Vernon, and Lee (2000) correlated brain volume by means of MRI in 68 adults and found *r* = 0.38 with *g* extracted from a battery of cognitive tests, but 0.59 when using correlated vectors. Wickett et al.'s head perimeter measure similarly went from 0.19 to 0.34. Schoenemann et al. (2000) obtained a simple correlation of 0.45 between brain volume and *g*, which Jensen (1998, p. 147) found to be 0.51 using the method of correlated vectors. Jensen (personal communication, August 8, 2002) carried out a vector analysis of the MRI study by MacLullich et al. (2002) and raised the correlation between *g* and cognitive ability from 0.42 to 0.78. Finally, Colom et al. (2006a) reanalyzed published data on 47 adults and found a correlation of 0.89 between *g* loadings and number of gray matter clusters, which was higher than the baseline correlations of 0.28–0.51 (Haier, Jung, Yeo, Head, & Alkire, 2004; Wilke, Sohn, Byars, & Holland, 2003).

Additional findings in Appendix 1 are of interest. For example, the brain volume–GMA correlation is equally strong in males and females (e.g., Andreasen et al., 1993; Wickett, Vernon, & Lee, 1994, 2000). It is also found in people of East Asian, East Indian, European, Turkish, African, South American, and Amerindian descent (e.g., Andreasen et al., 1993; Ivanovic et al., 2004; Tan et al., 1999). Age, although it plays a role in brain size and GMA, does not confound the results. Studies using a narrow age range of younger or older samples show the same magnitude of correlation (e.g., Deary et al., 2007; Egan et al., 1994).

Several studies have examined whether different regions of the brain would show differential correlations with GMA. Many appear to show that the size effects are manifest throughout the brain and not specific to any particular region (Andreasen et al., 1993; Reiss et al., 1996). However, other studies show GMA centered in the frontal brain regions (Jung & Haier, 2007). Colom et al. (2006a, 2006b) used the method of correlated vectors and found evidence for both positions—the more *g*-loaded subtests were distributed throughout the brain but concentrated most in the frontal lobes.

A unique study that did not fit the appendix is Witelson et al.'s (Witelson, Beresh, & Kigar, 2006) prospective study of 100 cancer patients (58 women and 42 men) who completed the Wechsler Adult Intelligence Scale and agreed to a postmortem examination. The subjects were Caucasian 25- to 83-year-olds (mean = 56 years). Although the subjects were ambulatory and well functioning, they were all on medication, which may have affected their performance. Also, some patients survived for several years after completing the tests, resulting in a delay before brain measurement. Nonetheless, the average correlation between brain weight and GMA for males was *r* = 0.26, and for females it was *r* = 0.31.

A functional relation between brain size and cognitive ability has been found in four studies showing that the correlation between brain size and GMA holds true *within* families as well as *between* families (Bergvall et al., 2006; Gignac et al., 2003; Jensen, 1994; Jensen & Johnson, 1994; although one study failed to do so: Schoenemann et al., 2000). The within-family finding is of special interest because it controls for most of the sources of variance that distinguish families, such as social class, styles of child rearing, and general nutrition, that differ between families. The largest of these is a population-based study that measured head size at birth and GMA at age 18 at the time of conscription in the Swedish military (Bergvall et al., 2006). Analyses were made of data on 96,189 males who had at least one full brother similarly measured. The between-family analysis showed that a small head circumference for gestational age (>1 SD below the mean) was associated with a 10% increase in risk of low intellectual performance (the lowest 11% of scores); the within-family analysis showed a 5% increase in risk.

The human brain may contain up to 100 billion (10^11^) nerve cells classifiable into 10,000 types resulting in 100,000 billion synapses (Kandel, 1991). The number of neurons available to process information may mediate the correlation between brain size and GMA. Haug (1987, p. 135) showed a correlation of *r* = 0.48 (*N* = 81, *p* < 0.001) between number of cortical neurons (based on a partial count of representative areas of the brain) and brain size. The regression equation was number of cortical neurons (in billions) = 5.583 + 0.006 (cm^3^ brain volume). The difference between the low end of normal (1,000 cm^3^) and the high end (1,700 cm^3^) worked out to be 4.283 billion neurons. Subsequently, Pakkenberg and Gundersen (1997) found a correlation of *r* = 0.56 between brain size and number of neurons.

Brain size is also correlated with body size. Results from autopsy studies such as the one by Dekaban and Sadowsky (1978) of 2,773 men and 1,963 women, as well as the one by Ho, Roessmann, Straumfjord, and Monroe (1980) of 644 men and 617 women, suggest a correlation of about 0.20 between brain mass (grams) and stature and body mass. Witelson et al.'s (2006) study of 100 cases found body size accounted for from 1% to 4% of the variation in cerebral volume in each sex. Similarly, MRI studies find an average correlation of about 0.20 (Pearlson et al., 1989; Wickett et al., 1994). The relationship is higher (0.30–0.40) with measures of the skull (cm^3^), estimated either from endocranial volume or from external head measures. In a stratified random sample of 6,325 U.S. servicemen, cranial capacity correlated, on average, 0.38 with height and 0.41 with mass in 2,803 women and 3,522 men (Rushton, 1992a).

Body size is also correlated with GMA about 0.20–0.25. A study of 10,424 children born in Aberdeen (Scotland) between 1950 and 1956 found correlations of *r* = 0.20–0.25 between body weight at birth and GMA at age 7 years and between height at age 7 and GMA at 11 years (Lawlor et al., 2005). A nation-wide study of 950,000 Swedish men, with analyses made of full-brother-pairs to control for the effects of shared family environment, found a significant relation between heights at age 18 years and completed education at 27 years (Magnusson, Rasmussen, & Gyllensten, 2006).

There is disagreement about whether or not brain size should be corrected for body size when examining brain size/GMA correlations. Controlling for body size changes the question from “Is IQ correlated with absolute brain size?” to “Is IQ correlated with relative brain size?” Although these are quite different questions, evidence shows that the answer to both is yes.

The brain size/GMA relation is also found in other species. For example, Hamilton (1935) found that rats selected for 12 generations to be either “maze-bright” or “maze-dull” differed by 2.5 standard deviations in brain weight; within unselected control rats, there was a correlation of *r* = 0.25 between maze ability and brain weight. Anderson (1993) found a correlation of *r* = 0.48 in rats between brain weight at autopsy and GMA extracted from several cognitive tasks. Madden (2001) found that species of bowerbirds that build more complex bowers have larger brains than species that build less complex ones. Schultz, Bradbury, Evans, Gregory, and Blackburn (2005) found that bird species with bigger brains (such as blue tits and magpies) adapted better to the changing British farm and urban environment and were reproductively more successful than populations of birds with smaller brains (like gray partridges and corn buntings). Dunbar and Shultz (2007) found among 24 primate species that the size of the neocortex (relative to body size) correlated *r* = 0.67 with the size of the social group. Also across 24 primate species, Deaner et al. (2007) found that whole brain size correlates *r* = 0.65 with domain general learning ability.

### Reaction Time Measures

Reaction times (RTs) provide increasingly good measures of *g* (Jensen, 2006). This research too began with Francis Galton who included them as part of the battery of tests he administered in his Anthropometic Laboratory in London's South Kensington Museum. From 1884 to 1890, Galton obtained data from more than 10,000 men, women, and children. Galton (1883) found, as others have since, that RTs are simpler to devise and administer than psychometric tests. Unfortunately, the results of the early studies appeared not to correlate with measures of IQ, in large part because of restriction of range, acondition not well known at the time, which leads to underestimating the correlations that would obtain in the general population. Consequently, Galton's procedures went out of fashion for many decades.

Reaction times are so easy to do that 9- to 12-year-old children can perform them in less than 1 s. On these simple tests, children with higher GMA scores perform faster than do children with lower scores, perhaps because reaction time measures the neurophysiological efficiency of the brain's capacity to process information accurately—the same ability measured by intelligence tests (Deary, 2000; Jensen, 2006). Children are not trained to perform well on reaction time tasks (as they are on certain paper-and-pencil tests), so the advantage of those with higher GMA scores on these tasks cannot arise from practice, familiarity, education, or training. Simple reaction time (SRT) measures correlate with IQ ∼ 0.20, while more complex choice reaction time (CRT) measures correlate ∼0.40. In aggregate, RTs can correlate 0.70 with IQ (Jensen, 2006).

Faster RTs also correlate with brain size measured by MRI (Wickett et al., 2000). The relationships between RT, brain size, and GMA may occur because they are all mediated by the neurophysiological efficiency of the brain's capacity to process information accurately (Deary, 2000; Jensen, 2006). Haier et al. (1995) tested the brain efficiency hypothesis by using MRI to measure brain volume and glucose metabolic rate to indicate energy use. They found a correlation of −0.58 between glucose metabolic rate and GMA, suggesting that more intelligent individuals have more efficient brains because they use less energy in performing a given cognitive task. Several other studies supporting the brain size/efficiency model were reviewed in Gignac et al. (2003). In any individual, however, energy use increases with the increasing complexity of the cognitive task.

## AGE DIFFERENCES

### Brain Size

**Figure 1:**
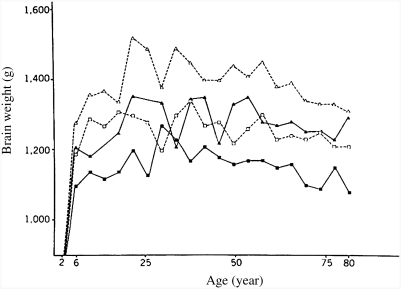
Mean brain weight for 4-year age periods in various subgroups. Brain weight is plotted at midpoint of each age period (e.g., the point at age 6 years represents the average for subjects between 4 and 8 years; White men, open triangles; Black men, solid triangles; White women, open squares; Black women, solid squares). Differences in brain weights among various groups become apparent at age 6 years. (From Ho et al., 1980, p. 636, Figure 2.)

Autopsy studies show that brain mass increases during childhood and adolescence and then, beginning as early as 20 years, slowly decreases through middle adulthood, and finally decreases more quickly in old age (Dekaban & Sadowsky, 1978; Ho et al., 1980; Pakkenberg & Voigt, 1964; Voigt & Pakkenberg, 1983). Broca first showed these relationships in the nineteenth century (see reanalysis by Schreider, 1966). The data of Ho et al. (1980), collated for 2,037 subjects from autopsy records, for various subgroups, 1,261 of them between the ages of 25 and 80, are shown in Figure 1. All brains were weighed on the same balance at the Institute of Pathology at Case Western Reserve University after excluding those brains with lesions or other abnormalities. The average mass of the brain increases from 397 g at birth to 1,180 g at 6 years. Growth then slows, and brain mass peaks at about 1,450 g before age 25 years. The mass declines slowly from age 26 to 80 at an average of 2 g per year. The decrease after age 80 years is much steeper, the loss being 5 g per year. As shown in Figure 1, although the rate of decrease varies slightly, it is essentially similar for various subgroups.

In a cross-sectional stereological investigation, which covered the age range from 20 years to 90 years, Pakkenberg and Gundersen (1997) found that approximately 10% of all neocortical neurons were lost over the life span in both sexes. MRI investigations also show a curvilinear pattern of growth and change, with an overall decrease in brain volume following the late teens as brain tissue is replaced with cerebrospinal fluid (CSF). For example, Pfefferbaum et al. (1994) demarcated cell growth, myelination, pruning, and atrophy, finding that from 3 months to 30 years, cortical gray matter volume (mainly cell bodies) peaked at around age 4 years and then declined steadily throughout the lifespan; cortical white matter volume (myelin sheath) increased steadily until about age 20 years and appeared stable thereafter; and cortical CSF remained stable from 3 months to 20 years and then increased exponentially from 21 to 71 years. Good et al. (2001) studied 465 men and women aged 17–75 years with no history of risk factors and found a decline of global gray matter volume with age of *r* = 0.49. In the Baltimore Longitudinal Study of Aging, 92 participants aged 59–85 years (at baseline) showed significant annual increases of 1,526 mm^3^ in ventricular volume (Resnick, Pham, Kraut, Zonderman, & Davatzikos 2003). In that study, volume loss was not uniform, and some areas of the brain reduced in size, while the volume of others was preserved. Staff, Murray, Deary, and Whalley (2006) found that the loss of brain volume in 79–80 year olds was associated with general declines in GMA rather than to any specific abilities.

### Cognitive Ability

General intelligence shows concomitant increases during childhood and adolescence and then (slow) decreases between ages 25 and 45, and (faster) decreases after age 45. For example, when David Wechsler (1944) normed the first Wechsler–Bellevue test of adult intelligence on a fairly representative sample of the adult population of the United States, he found that all 10 of the diverse verbal and nonverbal subtests given to successive age groups from 18 to 70 years of age showed an average decline in test scores with increasing age. Wechsler wrote:

We have put forward the hypothesis that the decline of mental ability with age is part of the general organic process which constitutes the universal phenomenon of senescence, and have insisted upon the fact that the phenomenon begins relatively early in life. The evidence we have adduced for this hypothesis is the observed parallelism we have found in the decline of various physical and mental abilities.

It once was claimed that this age-related decline in GMA was spurious because early longitudinal studies contradicted findings from cross-sectional studies; thus, the cross-sectional observations were derogated as a generation or “cohort” effect, perhaps due to “more favorable” environments for younger cohorts. However, several subsequent longitudinal studies, reviewed by Brody (1992) and Deary (2000), have corroborated the results from the cross-sectional studies. Brody (1992, p. 238) concluded, “Declines in fluid ability over the life span up to age 80 might well average 2 standard deviations.”

### Reaction Time Measures

Reaction time tasks also show the decline with age. The most definitive evidence comes from two population representative studies. Deary and Der (2005) tested 500+ 16-, 36-, and 56-year-olds from the West of Scotland. Participants were retested 8 years later, at which time they also took the *g*-loaded Paced Auditory Serial Addition Test (PASAT). Individual differences on the RT measures were stable over the 8-year period (*r* ∼ 0.50), correlated with the PASAT scores (mean *r* ∼ 0.25), and declined with age (CRT from age 20; SRT from age 50). Subsequently, Der and Deary (2006) reanalyzed data for 7,130 adults in the UK's Health and Lifestyle Survey. Again, CRT declined from age 20 and SRT from age 50.

## SOCIOECONOMIC POSITION DIFFERENCES

### Brain Size

Nineteenth- and early twentieth-century data from Broca (1861) and others (Hooton, 1939; Sorokin, 1927; Topinard, 1878) suggested that people in higher-status occupations averaged a larger brain or head size than those in lower ones. Galton (1883) collected head size measures and information on the educational and occupational background of thousands of individual visitors to the South Kensington Natural Science Museum in London. However, he had no statistical method for testing the significance of the differences in head size between various occupational or educational groups. Nearly a century later, Galton's data were analyzed by Johnson et al. (1985), who found that professional and semiprofessional groups averaged significantly larger head sizes (in both length and width) than unskilled occupational groups. Subsequently, Rushton and Ankney (1996) calculated cranial capacities from the summary by Johnson et al. (1985) of Galton's head size data and found that cranial capacity increased from unskilled to professional classes from 1,324 to 1,468 cm^3^ in men and from 1,256 to 1,264 cm^3^ in women. These figures are uncorrected for body size.

The relationship between head size and occupational status has also been found after correcting for body size. Jensen and Sinha (1993) reviewed much of the literature. They drew an important distinction between a person's socioeconomic position (SEP) of origin (the SEP attained by the person's parents) and the individual's attained SEP (the SEP attained by the person in adulthood). Correlations of IQ, head size, and other variables are always smaller when derived from the SEP of origin than when derived from attained SEP. Thus, Jensen and Sinha analyzed the head circumference data from the National Collaborative Perinatal Project (Broman et al., 1975) of approximately 10,000 White and 12,000 Black 4-year-old children and found a small but significant correlation with social class of origin within both the White and Black populations, after height was controlled for (*r* = 0.10). Jensen and Sinha also reanalyzed autopsy data reported by Passingham (1979) on 734 men and 305 women and found an overall correlation between brain mass and achieved occupational level of about 0.25, independent of body size.

Studies using brain imaging techniques have also reported significant main effects of brain size on occupational status and education level; higher-status subjects had, on average, a larger brain than lower-status subjects (Andreasen et al., 1990; Pearlson et al., 1989). Rushton (1992a) used the externally measured cranial size of 6,325 U.S. servicemen and found that officers averaged significantly larger cranial capacities than enlisted personnel either before or after adjusting for the effects of stature, weight, race, and sex (1,384 vs. 1,374 cm^3^ before adjustments; 1,393 vs. 1,375 cm^3^ after adjustments). The differences between officers and enlisted personnel were found for both men and women, as well as for East Asians, Whites, and Blacks.

### Cognitive Ability

IQ test scores are significantly correlated with the socioeconomic hierarchies of modern Europe, North America, and Japan (Herrnstein & Murray, 1994; Jensen, 1998). The basic finding is that there is a difference of nearly 3 SD (45IQ points) between average members of professional and unskilled classes. These are group mean differences with considerable overlap of distributions. Nonetheless, the overall correlation between an individual's IQ and his or her SEP of origin is between 0.30 and 0.40, and the correlation between IQ and attained SEP, or occupational level, is about 0.50 (Herrnstein & Murray, 1994). A study of nearly 15,000 children born between 1950 and 1956 replicated the relationship with measures made of father's SEP at the time of the child's birth and the child's GMA at ages 7, 9, and 11 years (Lawlor et al., 2005). Even after adjustment for maternal characteristics and perinatal factors, father's SEP accounted for 6% of the variation in child's GMA at age 11.

In studies of intergenerational social mobility, Mascie-Taylor and Gibson (1978) and Waller (1971) obtained GMA scores of fathers and their adult sons. They found that, on average, children with lower test scores than their fathers had gone down in SEP as adults, but those with higher test scores had gone up. A within-family study was also conducted by Murray (1998), who found that among the 1,074 sibling pairs in the National Longitudinal Survey of Youth who had taken the Armed Forces Qualification Test, the sibling with higher GMA achieved a higher level of education, a higher occupational status, and greater take home pay than the sibling with lower GMA.

## SEX DIFFERENCES

### Brain Size

An absolute difference in average brain size between men and women has not been disputed since at least the time of Broca (1861). It is often claimed, however, that this difference disappears when corrections are made for body size or age of people sampled (Gould, 1981, 1996). However, Ankney (1992) demonstrated that the sex difference in brain size remains after corrections for body size in a sample of similarly aged men and women (following tentative results by Dekaban & Sadowsky, 1978; Gur et al., 1991; Hofman & Holloway, 1980; Swaab, 1991; Swaab & Hofman, 1984; Willerman et al., 1991).

**Figure 2:**
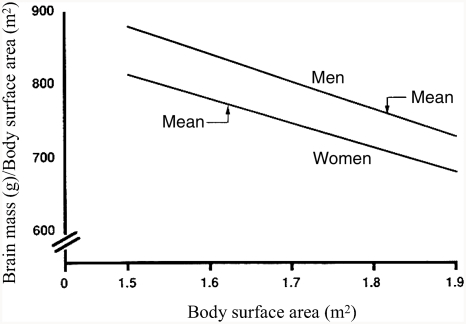
The relation between the ratio of brain mass/body surface area and body surface area in White men and women. Ankney (1992) calculated the ratios by estimating brain mass at a given body surface area using the equations in Ho et al. (1980, Table 3): *men*, brain mass = 1,077 g (±56) +173 (±31) × body surface area (*r* = +0.27, *p* < 0.01); *women*, brain mass = 949 g (±52) +188 (±32) × body surface area (*r* = +0.24, *p* < 0.01). (From Ankney, 1992, p. 331, Figure 1. Copyright 1992 by Ablex Publishing Corp. Reprinted with permission.).

Ankney (1992) suggested that the large sex difference in brain size went unnoticed for so long because earlier studies used improper statistical techniques to correct for sex differences in body size and thus incorrectly made a large difference “disappear.” The serious methodological error was the use of brain mass/body size ratios instead of analysis of covariance (see Packard & Boardman, 1988). Ankney (1992) illustrated why this is erroneous by showing that, in both men and women, the ratio of brain mass to body size declines as body size increases. Thus, as can be seen in Figure 2, larger women have a lower ratio than smaller women, and the same holds for larger men compared with smaller men. Therefore, because the average-sized man is larger than the average-sized woman, their brain mass to body size ratios are similar. Consequently, the only meaningful comparison is that of brain mass to body size ratios of men and women of equal size. Such comparisons show that at any given size, the ratio of brain mass to body size is much higher in men than in women (Figure 2).

**Figure 3:**
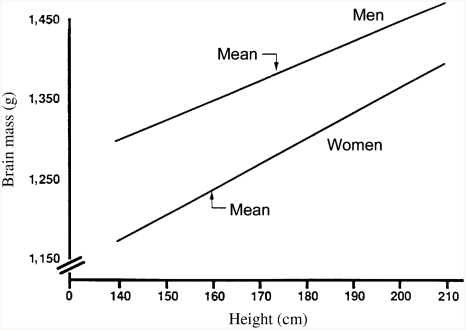
The relation between brain mass and body height in White men and women. Lines drawn from equations in Ho et al. (1980, Table 1): *men*, brain mass = 920 g (±113) + 2.70 (±0.65) × body height (*r* = 0.20, *p* < 0.01); *women*, brain mass = 748 g (±104) + 3.10 (±0.64) × body height (*r* = +0.24, *p* < 0.01). (From Ankney, 1992, p. 333, Figure 4. Copyright 1992 by Ablex Publishing Corp. Reprinted with permission.).

Ankney (1992) reexamined autopsy data on 1,261 American adults (Ho et al., 1980) and found that at any given body surface area or height, brains of White men are heavier than those of White women, and brains of Black men are heavier than those of Black women. For example, among Whites 168 cm (5′7″) tall (the approximate overall mean height for men and women combined), brain mass of men averages about 100 g heavier than that of women (Figure 3), whereas the average difference in brain mass, uncorrected for body size, is 140 g. Thus, only about 30% of the sex difference in brain size is due to differences in body size.

**Figure 4:**
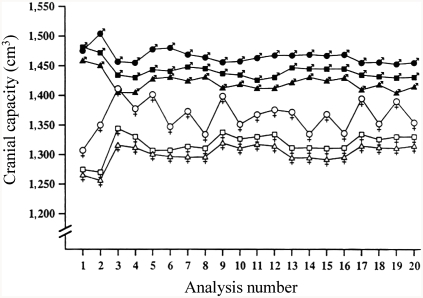
Cranial capacity for a stratified random sample of 6,325 U.S. Army personnel. The data, grouped into six sex-by-race categories, are collapsed across military rank. (East Asian men, closed circles; White men, closed squares; Black men, closed triangles; East Asian women, open circles; White women, open squares; Black women, open triangles). They show that, across the 19 different analyses controlling for body size, men averaged larger cranial capacities than did women, and East Asians averaged larger than did Whites or Blacks. Analysis 1 presents the data unadjusted for body size showing no difference for East Asian and White men. (From Rushton, 1992a, p. 408, Figure 1. Copyright 1992 by Ablex Publishing Corp. Reprinted with permission.).

Ankney's results were confirmed in a study of cranial capacity in a stratified random sample of 6,325 U.S. Army personnel (Rushton, 1992a). After adjusting, by means of analysis of covariance for effects of age, stature, weight, military rank, and race, men averaged 1,442 cm^3^ and women 1,332 cm^3^. This difference was found in all of the 20 or more separate analyses (shown in Figure 4) conducted to rule out any body size effect. Moreover, the difference was replicated across samples of East Asians, Whites, and Blacks, as well as across officers and enlisted personnel. Parenthetically, in the army data, East Asian women constituted the smallest sample (*N* = 132), and it is probable that this caused the “instability” in estimates of their cranial size when some corrections were made for body size (Figure 4). The sex difference of 110 cm^3^ found by Rushton, from analysis of external head measurements, is remarkably similar to that (100 g) obtained by Ankney, from analysis of brain mass (1 cm^3^ = 1.036 g; Hoffman, 1991).

Studies using MRI have also confirmed the sex difference in adult brain size (Good et al., 2001; Harvey et al., 1994; Reiss et al., 1996; Willerman et al., 1991). Thus, Ivanovic et al. (2004) carried out a study that controlled for body size in 96 18-year-old male and female high school graduates in Chile and found that the males averaged 1,480 cm^3^ (SD = 125) before body size adjustments and 1,470 cm^3^ (SD = 40) after adjustments, while the females averaged 1,394 cm^3^ (SD = 89) before and 1,404 cm^3^ (SD = 37) after adjustments.

A stereological investigation by Pakkenberg and Gundersen (1997) found that men had about 4 billion more cortical neurons than did women, and this was not accounted for by differences in height. The average number of neocortical neurons was 19 billion in female brains and 23 billion in male brains, a 16% difference. In their study, which covered the age range from 20 years to 90 years, approximately 10% of all neocortical neurons were lost over the life span in both sexes. Sex and age were the main determinants of the total number of neurons in the human cortex, whereas body size per se had no influence on neuron number.

From birth through the early months, Rushton and Ankney (1966) found the sex difference held across several autopsy studies when, following Ankney's (1992) procedure (see Figure 3), brain masses of boys and girls were compared after matching them for stature (Dekaban & Sadowsky, 1978; Pakkenberg & Voigt, 1964; Voigt & Pakkenberg, 1983). At birth, boys averaged a cranial capacity 5 cm^3^ larger than girls, a difference that increased to 40 cm^3^ by 4 months and 50 cm^3^ by age 1 year, and then remained stable through to age 7 years (Rushton, 1997; controlling for body size). From 7 to 17 years, sex differences in cranial capacity were in the range of 60–100 cm^3^ (Lynn, 1993; Rushton & Osborne, 1995).

### Cognitive Ability

The sex differences in brain size present a paradox. Women have proportionately smaller average brains than men but apparently have the same intelligence test scores. According to Kimura (1999), women excel in verbal ability, perceptual speed, and motor coordination within personal space, whereas men do better on various spatial tests and on tests of mathematical reasoning. A review by Voyer et al. (1995) showed that on the “purest” spatial measures, such as rotating an imaginary object or shooting at a moving rather than a stationary target, the sex difference approaches 1 SD. Ankney (1992, 1995) therefore hypothesized that the sex difference in brain size relates to those intellectual abilities at which men excel; that is, spatial and mathematical abilities require more “brain power.” Analogously, whereas increasing word-processing power in a computer requires some extra capacity, increasing three-dimensional processing, as in graphics, requires a major increase in capacity.

Unfortunately for this hypothesis, what little information there is from the two MRI studies to date suggests that brain size is not significantly related to results on purely spatial tests (such as mental rotation) in either men or women (Wickett et al., 1994, 2000). Yet in the same studies, brain size did correlate significantly with IQ. However, one of these studies looked at only women and the other looked at only men. It would be more informative to know what happens in a *combined* sample of men and women, since the hypothesis that the extra brain size relates to men's better spatial scores would predict a correlation that should appear across sexes. So far, no comparison of brain size and spatial scores has been made in a mixed-sex group.

Richard Lynn (1994, 1999) provided a resolution of the “the Ankney–Rushton anomaly” (1999, p. 1) of sex differences in brain size by resurrecting the nineteenth century proposition that men average slightly higher in general intelligence than women (e.g., Broca, 1861, p. 153). He reviewed data from Britain, Greece, China, Israel, the Netherlands, Norway, Sweden, Japan, India, and Indonesia, as well as the United States to show that men averaged about 4 IQ points higher than women on a number of published tests. He noted that age is an important variable because the male advantage in GMA does not emerge until the late adolescent growth spurt when brain size differences peak. Girls mature faster than boys, which give them an early advantage in language development and may mask later cognitive differences. Lynn suggested that this may have led generations of researchers, who relied on school samples, to miss the later emerging sex difference. Subsequently, in meta-analyses of general population samples on the Standard and Advanced Progressive Matrices, Lynn and Irwing (2004; Irwing & Lynn, 2005, 2006) found no sex difference among children aged 6–14 years but a male advantage from 15 years through old age. They found that by adulthood, the male advantage is equivalent to between 3.3 and 5.0 IQ points, with 4.6 being the best estimate.

Other researchers have corroborated Lynn's results (Jackson & Rushton, 2006; Nyborg, 2003, 2005). For example, Jackson and Rushton (2006) analyzed data from 100,000 17- and 18-year-olds who had completed the 145-item Scholastic Assessment Test (SAT). The *g* factor was found to underlie both the SAT Verbal (SAT-V) and the SAT Mathematics (SAT-M) scales and to be a better predictor of student grades than the traditionally used SAT-V and SAT-M scales. The male and female *g* factors were congruent in excess of 0.99 and found to favor males with an effect size of 0.24, equivalent to 3.63 IQ points. The male advantage was found throughout the entire distribution of scores on the SAT, in every level of family income, for every level of fathers' and mothers' education, and for each and every one of seven ethnic groups.

### Reaction Time Measures

Reaction time tasks show a commensurate male advantage with effect sizes of *d* = 0.17 to 0.40 for simple and choice RTs, respectively. In a meta-analysis of 72 effect sizes derived from 21 studies (*N* = 15,003) of SRT, Silverman (2006) found an effect size favoring men of 0.17. Deary and Der (2005) found a male advantage of *d* ∼ 0.40 on CRT measured from a representative sample of 500+ Scottish 16- to 56-year-olds. The men also scored higher on the *g*-loaded Paced Auditory Serial Addition Test (*d* ∼ 0.20). In a representative sample of 7,130 adults in the UK's Health and Lifestyle Survey, Der and Deary (2006) again found men consistently averaged faster RTs.

## POPULATION GROUP DIFFERENCES

### Brain Size

Even before birth, population group differences in average brain size are found from the ninth week of intrauterine life. Schultz (1922) found that 455 White fetuses averaged larger brain cases and smaller faces than did 168 Black fetuses, with the differences becoming more prominent over the course of fetal development. Other traits were found, with Black fetuses averaging a longer forearm to upper arm ratio, a characteristic of Black adults, and a longer fourth to second finger ratio, also characteristic of Black adults (Manning, 2002).

**Figure 5:**
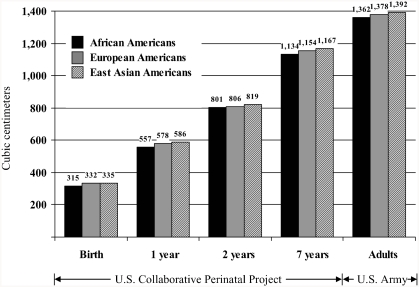
Mean cranial capacity (cm^3^) for African Americans, European Americans, and East Asian Americans from birth through adulthood. Data for birth through age 7 years from the U.S. Perinatal Project; data for adults from the U.S. Army data in Figure 4. (From Rushton, 1997, p. 15, Figure 2. Copyright 1997 by Ablex Publishing Corp. Reprinted with permission.).

Rushton (1997) analyzed population group differences from birth to age 7 years using measurements of head circumference and GMA gathered on 40,000 children by the U.S. Collaborative Perinatal Project (Broman et al., 1987). The results showed that at birth, 4 months, 1 year, and 7 years, the East Asian children averaged larger cranial volumes than White children who averaged larger cranial volumes than Black children (Figure 5). Within each group, children with larger head sizes obtained higher IQ scores. Moreover, the East Asian children, who averaged the largest craniums, were the shortest in stature and the lightest in weight, whereas the Black children, who averaged the smallest craniums, were the tallest in stature and the heaviest in weight; the differences in brain size were not due to body size.

Dozens of studies from the 1840s to the 1990s, using four different methods of measuring brain size—MRI, endocranial volume measured from empty skulls, wet brain weight at autopsy, and external head size measurements—all yield similar results. Using MRI, for example, Harvey et al. (1994) found that 41 Africans and West Indians in the United Kingdom had a smaller average brain volume than 67 Caucasians, although Harvey et al. provided no details on how, or if, the samples were matched for age, sex, or body size. In another British study, Jones et al. (1994) found a (not significant) trend for Whites to average a 30 cm^3^ larger intracranial volume and smaller ventricles than Afro-Caribbeans.

Measuring endocranial volume, the American anthropologist Samuel George Morton (1849) filled over 1,000 skulls with packing material and found that Blacks averaged about 5 cubic inches less cranial capacity than Whites. These results have stood the test of time (Gordon, 1934; Simmons, 1942; Todd, 1923). The largest study of race differences in endocranial volume was by Beals et al. (1984) with measurements of up to 20,000 skulls from around the world. They found that East Asians, Europeans, and Africans averaged cranial volumes of 1,415, 1,362, and 1,268 cm^3^, respectively.

Weighing brains at autopsy, Broca (1873) found that Whites averaged heavier brains than Blacks and had more complex convolutions and larger frontal lobes. Subsequent studies have found an average Black–White difference of about 100 g (Bean, 1906; Mall, 1909; Pearl, 1934; Vint, 1934). Some studies have found that the more White admixture (judged independently from skin color), the greater the average brain weight in Blacks (Bean, 1906; Pearl, 1934). In a study of 1,261 American adults, Ho et al. (1980) found that 811 White Americans averaged 1,323 g and 450 Black Americans averaged 1,223 g (Figure 1). Since the Blacks and Whites were similar in body size, differences in body size cannot explain away the differences in brain weight.

The largest autopsy study, as yet unpublished, is by anthropologist Ralph Holloway at Columbia University Medical School (personal communications, March 16, 2002, August 26, 2004). He found that in both men and women aged 18–65 years, 615 Blacks, 153 Hispanics, and 1,391 Whites averaged brain weights of 1,222, 1,253, and 1,285 g, respectively. The population groups were all of similar body size. There were also a large number (*N* = 5,731) of brain weights from 15- to 50-year-old Chinese from Hong Kong and Singapore that averaged 1,290 g.

Cranial volume has also been estimated from external head size measurements (length, width, height). For example, Rushton (1991) examined head size measures in 24 international military samples collated by the U.S. National Aeronautics and Space Administration (NASA) and, after adjusting for the effects of body height, weight, and surface area, found cranial capacity for East Asians was 1,460 cm^3^ and for Europeans, 1,446 cm^3^. Rushton (1992a) also calculated average cranial capacities for East Asians, Whites, and Blacks from a stratified random sample of 6,325 U.S. Army personnel and found an average of 1,416, 1,380, and 1,359 cm^3^, respectively. This study allowed precise adjustments for all kinds of body size measures. Yet adjusting for these did not erase the differences in cranial capacity.

### Cognitive Ability

Population group differences in measured intelligence parallel those found in brain size (Jensen, 1998; Lynn, 2006; Rushton & Jensen, 2005). East Asians assessed in North America and in Pacific Rim countries average IQs in the range of 101–111, with a mean of 106. Europeans in North America, Europe, Australasia, and South Africa average an IQ of between 85 and 115, with a mean of 100. African-descended people in North America, the Caribbean, and Europe, as well as in Africa, average a mean IQ of from 70 to 90.

Questions remain about the validity of using IQ tests for population group comparisons. However, because the tests show similar patterns of internal item consistency and predictive validity for all groups, and because the same differences are found on relatively culture-free tests, many have concluded that the tests *are* valid (Jensen, 1998; Lynn, 2006; Neisser et al., 1996). For example, Sternberg et al. (2001) found that GMA in Kenyan 12- to 15-year-olds predicted school grades at the same levels as they do in the West (mean *r* = 0.40, *p* < 0.001). Rushton, Skuy, and Bons (2004) found that GMA predicted university performance equally well in African and non-African engineering students (*r* ∼ 0.30; *p* < 0.05). Rindermann (2007) found the IQ estimates from countries around the world correlate with those country's test scores in mathematics and school achievement.

### Reaction Time Measures

The same three-way pattern of race differences has been found using the simplest culture-free cognitive measures such as reaction time tasks, which 9- to 12-year-old children perform in less than 1 s. Lynn (2006) found that East Asian children from Hong Kong and Japan were faster than European children from Britain and Ireland, who in turn were faster than African children from South Africa. Using similar tasks, this pattern of racial differences was also found in California (Jensen, 1998; Rushton & Jensen, 2005). Within each group, the children with higher IQ scores perform faster those with lower scores.

## EVOLUTION AND GENETICS

### Evolution

**Figure 6:**
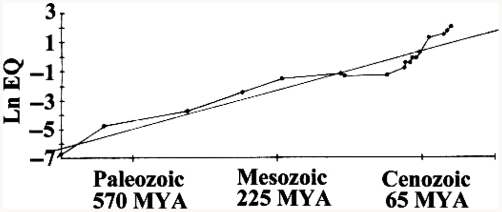
Average encephalization quotient (EQ; natural log), a measure of neural tissue corrected by body size, plotted against elapsed geologic time in millions of years. (After Russell, 1983).

It is reasonable to hypothesize that bigger brains have evolved based on natural selection for increased intelligence (Darwin, 1871; Geary, 2005; Jerison, 1973). Over the last 575 million years of evolutionary history, neural complexity and brain size have increased in vertebrates and invertebrates alike (Figure 6), little of which can be explained by body size increases. Russell (1983) calculated encephalization quotients, or EQs, a measure of actual brain size to expected brain size for an animal of that body weight [following Jerison, 1973; EQ = Cranial capacity (cm^3^)/(0.12)(body weight in grams)^0.67^]. Russell (1983) found that the mean EQ was only about 0.30 for mammals living 65 million years ago compared to the average of 1.00 today. EQs for molluscs varied between 0.043 and 0.31, and for insects between 0.008 and 0.045, with the less encephalized species resembling forms that appeared early in the geologic record and the more encephalized species resembling those that appeared later.

Metabolically, the human brain is an expensive organ. Representing only 2% of body mass, the brain uses about 5% of basal metabolic rate in rats, cats, and dogs, about 10% in rhesus monkeys and other primates, and about 20% in humans (Peters et al., 2004). Moreover, as large brains evolved, they required more prolonged and complex life histories to sustain them. For example, across 234 mammalian species Rushton (2004) found that brain weight correlated with longevity (*r* = 0.70), gestation time (0.72), birth weight (0.44), litter size (−0.43), age at first mating (0.63), duration of lactation (0.62), body weight (0.44), and body length (0.54). Even after controlling for body weight and body length, brain size continued to predict the other variables (*r* = 0.59). From an adaptationist perspective, unless large brains substantially contributed to evolutionary fitness (defined as increased survival of genes through successive generations), they would not have evolved. In the evolutionary competition to find and fill new niches, there is always “room at the top” for larger brain size and greater behavioral complexity.

The sexual dimorphism in cranial size and cognitive ability likely originated partly through evolutionary selection of men's hunting ability (Ankney, 1992; Kolakowski & Malina, 1974) and partly through the reproductive success socially dominant men have traditionally enjoyed (Geary, 2005; Lynn, 1994). Population–group differences may have originated from evolutionary pressures presented in colder climates (Rushton, 1995; Templer & Arikawa, 2006). Of course, brain size and intellectual performance are also affected by nutrition and experience (Flynn, 2007; Sternberg, 2004).

### Genetics

Heritabilities of 50%–80% are found for both brain size and GMA as well as the relation between them. For example, Bouchard and McGue (2003) reviewed data on more than 10,000 pairs of identical and same-sex fraternal twins living together and found the mean correlations were 0.86 and 0.60, respectively. They also found that for identical twins reared apart, the correlation was almost as high as for identical twins reared together (*r* = 0.78 for 93 pairs). For more than 27,000 pairs of nontwin siblings living together, the correlation was 0.49. In an MRI study of 112 extended twin families, Posthuma et al. (2002) found heritabilities of 82% for whole-brain gray matter volume, 87% for whole-brain white matter volume, and 86% for GMA.

Detailed three-dimensional brain maps reveal how brain structure is influenced by individual genetic differences. In a study of 10 identical and 10 same-sex fraternal twin pairs (*N* = 40), Thompson et al. (2001) found a genetic continuum in which brain structure was increasingly similar in subjects with increasing genetic affinity. Genetic factors were most marked in cortical structures in Broca's and Wenicke's language areas, as well as in frontal brain regions, which appeared to mediate differences in GMA in this study.

Evidence suggests that the age, SEP, sex, and population group differences are at least partly heritable because the heritabilities are about the same magnitude in all groups (Jensen, 1998; Plomin, DeFries, McClearn, & McGuffin, 2001). Moreover, Rushton, Bons, et al. (2007) estimated the heritability of scores on the diagrammatic puzzles of the Raven's Matrices, a culture-reduced test of GMA, from data on the identical twins from the Minnesota Study of Twins Reared Apart and found the differences between East Asians, Europeans, South Asians, and Africans were all more pronounced on the more heritable items (mean *r* = 0.40; *Ns* = 58; *p* < 0.05). This finding implies at least some genetic causation for the group differences. However, more definitive evidence will require the identification of the specific genes involved, although such evidence has been slow in coming (Plomin, Kennedy, & Craig, 2006).

Two newly discovered genes, *Microcephalin (MCPH1)* on chromosome 8p23 and abnormal spindle-like microcephaly associated (*ASPM*) on chromosome 1q31, attracted much attention when reported to be (1) associated with autosomal recessive primary microcephaly, (2) positively accelerated in molecular evolutionary rate through the simian line leading to *Homo sapiens*, and (3) under recent positive selection in modern humans (Evans et al., 2005; Mekel-Bobrov et al., 2005). The *MCPH1* allele favored by selection in modern humans, known as the D (derived) allele, was estimated to have arisen approximately 37,000 years ago (95% CI: 60,000–14,000 years ago), about the time symbolic behavior became widespread in Europe, while the favored D allele for *ASPM* was estimated to have arisen approximately 5,800 years ago (95% CI: 14,000–500 years ago), about the time cities developed in the Near East. Both genes were hypothesized to confer a selective advantage such as increased brain size and GMA.

Unfortunately for the *Microcephalin* hypothesis, four sets of association studies have now found normal variants of these genes are unrelated to either brain size or GMA (Dobson-Stone et al., 2007; Mekel-Bobrov et al., 2007; Rushton, Vernon, et al., 2007; Woods et al., 2006). For example, Rushton, Vernon, et al. (2007) found no relation in 644 Canadian subjects with two tests of GMA, a measure of head circumference, and two tests of social intelligence (altruism and prosocial attitudes).

## CONCLUSION

The preponderance of evidence demonstrates that brain size is correlated positively with intelligence and that both brain size and GMA are correlated with age, socioeconomic position, sex, and population group differences. Correlation does not prove cause, but, just as zero correlations provide no support for a hypothesis of cause and effect, nonzero correlations do provide support. The brain size/GMA relation has been established both within and between species and brain size has shown a progressive trend upwards for 570 million years.

Brain size, of course, is also environmentally sensitive. For example, rats raised in complex environments have thicker cortices and larger brains than rats reared in impoverished environments (Diamond, 1988). This suggests that the direction of causality is bidirectional and complicated by gene–environment correlations and interactions. Genes for GMA likely cause individuals to experience more stimulating and complex environmental situations, thereby increasing their brain size and creating a “benign circle” between brain size and intellectual performance.

Numerous issues require much further research. Where exactly in the brain is GMA located and how is it mediated? Major advances here might soon be expected (Jung & Haier, 2007). Perhaps the most perplexing question is: where the genes are for brain size and GMA?Thousands of genes are expressed in the brain and null findings are common. Given that genetic effect sizes turn out to be extremely small, typically 0.1%, and contribute interchangeably and additively, most studies have been seriously underpowered to detect and replicate effects (Plomin et al., 2006). Association studies of many markers in thousands of individuals may be required to identify appropriate genes. Alternatively, just a few regulator genes may turn out to be crucial.

## Appendix 1. Brain volume relation to GMA using neurologically normal subjects

**Table:**

Source	Sample	GMA measure	Correlation
Willerman, Schultz, Rutledge and Bigler (1991)	20 European-American male university students with mean age = 18 years	WAIS-R	0.51
Willerman et al. (1991)	20 European-American female university students with mean age = 18 years	WAIS-R	0.33
Andreasen et al. (1993)	37 European-American males aged 18–75 years	WAIS-R	0.40
Andreasen et al. (1993)	30 European-American females aged 18–75 years	WAIS-R	0.44
Raz et al. (1993)	29 European-Americans (17 men, 12 women) with mean age = 43.8 years (SD = 21.5)	CFIT	0.43
Egan et al. (1994; corrected by Egan, Wickett, & Vernon, 1995)	40 British military (unreported sex and race breakdown) with mean age = 23 (SD = 5), corrected for height, weight, and restricted range	WAIS-R	0.48
Castellanos et al. (1994)	46 children aged 5–19 years of unknown background	WISC-R subscales	0.33
Harvey, Persaud, Ron, Baker, and Murray (1994)	34 healthy male and female British hospital staff and locals (62% Caucasian; 38% Afro-Caribbean) used as control group	NART	0.69
Jones et al. (1994)	67 healthy male and female British, aged 16–60 years, some Afro-Caribbean, used as a community control group	NAT or verbal subtest of the WAIS	0.30
Wickett et al. (1994)	40 White Canadian women aged 20–30 years; height and weight partialed out and corrected for restriction of range	MAB	0.40
Kareken et al. (1995)	68 Caucasian and non-Caucasian adults of both sexes aged 18–45 years	Average of various subtests	0.25
Reiss, Abrams, Singer, Ross, and Denckla (1996)	12 boys, mainly White, aged 5–17 years	WISC-R	0.52
Reiss et al. (1996)	57 girls, mainly White, aged 5–17 years	WISC-R	0.37
Flashman, Andreasen, Flaum, and Swayze (1998)	90 healthy normal volunteer controls (47% female) with mean age = 27 years (SD = 10)	WAIS-R	0.25
Tramo et al. (1998)	20 individuals (10 pairs of identical twins) aged 24–43 years; we use their total cortical surface area as the estimate of brain size	WAIS-R	0.20
Gur et al. (1999)	40 men with a mean age = 26 years (SD = 5.5)	Various	0.40
Gur et al. (1999)	40 women with a mean age = 26 years (SD = 5.5)	Various	0.39
Tan et al. (1999)	54 female university students in Turkey, aged 18–26 years	CFIT	0.62
Tan et al. (1999)	49 male university students in Turkey, aged 18–26 years	CFIT	0.28
Wickett et al. (2000)	68 individuals (34 pairs of brothers) aged 20–35 years	*g*, from MAB and other tests	0.38
Pennington et al. (2000)	96 individuals (48 pairs of MZ and DZ twins), mean age = 17 years (SD = 4.1)	WISC-R and WAIS-R	0.42
Pennington et al. (2000)	36 individuals (18 pairs of MZ and DZ twins), mean age = 19 years (SD = 3.7)	WISC-R and WAIS-R	0.31
Schoenemann, Budinger, Sarich, and Wang (2000)	72 individuals (36 pairs of sisters) aged 18–43 years	*g*, from 11 diverse cognitive tasks including Raven's Matrices with corrections for age	0.45
Aylward et al. (2002)	83 White men and women aged 8–46 years used as healthy controls	Unspecified IQ test	0.04
MacLullich et al. (2002)	97 healthy men aged 68 years (SD = 1.3)	*g*, from various tests including NART and Raven's Matrices	0.42
Ivanovic et al. (2004)	47 male 18-year-old high school students in Chile selected from the richest and poorest counties	WAIS-R	0.55
Ivanovic et al. (2004)	49 female 18-year-old high school students in Chile selected from the richest and poorest counties	WAIS-R	0.37
Deary et al. (2007)	48 male 71- to 76-year-olds resident in Scotland	NART	0.56
Number of samples: 28			
Total *N*: 1,389			
Unweighted mean *r* = 0.40			
*N*-weighted mean *r* = 0.38			

*Note*. CFIT, Culture-Free Intelligence Test; MAB, Multidimensional Aptitude Battery; MRI, Magnetic Resonance Imaging; NART, New Adult Reading Test; PMAT, Primary Mental Abilities Test; WAIS-R, Wechsler Adult Intelligence Scale-Revised; WISC, Wechsler Intelligence Scale for Children.

## Appendix 2. Head size relation to GMA using neurologically normal subjects

**Table:**

Study	Sample	Head size measure	GMA measure	Correlation
Pearl (1906)	935 German male soldiers	Perimeter	Officers' rating	0.14
Pearson (1906)	2,398 British boys aged 3–20 years, standardized to age 12	Length	Teachers' estimate	0.14
Pearson (1906)	2,188 British girls aged 3–20 years, standardized to age 12	Length	Teachers' estimate	0.08
Pearson (1906)	1,011 British male university students	Length	Grades	0.11
Murdock & Sullivan (1923)	291 American boys aged 6–17 years, standardized by age	Perimeter	Various IQ tests	0.20
Murdock & Sullivan (1923)	395 American girls aged 6 to 17 years, standardized by age	Perimeter	Various IQ tests	0.27
Reid & Mulligan (1923)	449 Scottish male medical students	Capacity	Grades	0.08
Sommerville (1924)	105 White male university students	Capacity	Thorndike	0.08
Estabrooks (1928)	172 White boys aged 7 years	Capacity	Binet IQ test	0.23
Estabrooks (1928)	207 White girls aged 7 years	Capacity	Binet IQ test	0.16
Porteus (1937)	200 White Australian children	Perimeter	Porteus Maze	0.20
Schreider (1968)	80 adult Otomi Amerindians from Mexico	Perimeter	Form board	0.39
Schreider (1968)	158 French farmers of unreported sex	Perimeter	Raven's Matrices	0.23
Klein, Freeman, Kagan, Yarborough, and Habicht (1972)	172 Guatemalan Amerindian boys aged 3–6 years	Perimeter	Knowledge tests, with age standardized	0.23
Klein et al. (1972)	170 Guatemalan Amerindian girls aged 3–6 years	Perimeter	Knowledge tests, with age standardized	0.29
Susanne & Sporcq (1973)	2,071 Belgian male conscripts	Perimeter	Raven's Matrices	0.19
Weinberg, Dietz, Penick, and McAlister (1974)	334 White boys aged 8–10 years	Perimeter	WISC	0.35
Passingham (1979)	415 English villagers (212 men, 203 women) aged 18–75 years	Capacity	WAIS	0.13
Susanne (1979)	2,071 Belgian male conscripts	Perimeter	Matrices	0.19
Pollitt, Mueller, and Liebel (1982)	91 boys and girls aged 3–6 years	Perimeter	Stanford-Binet	0.23
Majluf (1983)	120 boys and girls aged 8–20 months	Perimeter	Bayley motor development test	0.35
Ounsted, Moar, and Scott (1984)	214 boys aged 4 years	Perimeter	Language test	0.06
Ounsted et al. (1984)	167 girls aged 4 years	Perimeter	Language test	0.07
Henneberg, Budnik, Pezacka, and Puch (1985)	151 Polish male medical students aged 18–30 years	Capacity	Baley's Polish language IQ test	0.09
Henneberg et al. (1985)	151 Polish female medical students aged 18–30 years	Capacity	Baley's Polish language IQ test	0.19
Sen et al. (1986)	150 16- to 18-year-old males in India	Perimeter	Raven's Matrices	0.02
Sen et al. (1986)	150 16- to 18-year-old females in India	Perimeter	Raven's Matrices	0.54
Broman et al. (1987)	18,907 Black boys and girls aged 7 years	Perimeter	WISC	0.19
Broman et al. (1987)	17,241 White boys and girls aged 7 years	Perimeter	WISC	0.24
Ernhart, Marler, and Morrow-Tlucak (1987)	257 3-year-old boys and girls	Perimeter	Stanford-Binet	0.12
Bogaert & Rushton (1989)	216 White Canadian male and female university students, adjusted for sex	Perimeter	MAB	0.14
Lynn (1989)	161 Irish boys aged 9–10 years	Perimeter	PMAT	0.15
Lynn (1989)	149 Irish girls aged 9–10 years	Perimeter	PMAT	0.23
Lynn (1990)	205 Irish children aged 9 years	Perimeter	Raven's Matrices	0.26
Lynn (1990)	91 English children aged 9 years	Perimeter	Raven's Matrices	0.26
Osborne (1992)	106 European-American boys aged 13–17 years; controls for height and weight	Capacity	Basic	0.16
Osborne (1992)	84 African-American boys aged 13–17 years; controls for height and weight	Capacity	Basic	0.34
Osborne (1992)	118 European-American girls aged 13–17 years; controls for height and weight	Capacity	Basic	0.23
Osborne (1992)	168 African-American girls aged 13–17 years; controls for height and weight	Capacity	Basic	0.13
Rushton (1992b)	73 East Asian-Canadian male and female university students	Perimeter	MAB	0.14
Rushton (1992b)	211 White Canadian male and female university students	Perimeter	MAB	0.21
Lynn & Jindal (1993)	100 9-year-old boys from northern India	Perimeter	Matrices	0.14
Lynn & Jindal (1993)	100 9-year-old girls from northern India	Perimeter	Matrices	0.25
Reed & Jensen (1993)	211 European-American male college students	Capacity	Raven's Matrices	0.02
Wickett et al. (1994)	40 White Canadian female university students	Perimeter	MAB	0.11
Furlow, Armijo-Prewitt, Gangestad, and Thornhill, (1997)	128 undergraduates, (60% female; 52% Caucasian, 38% Hispanic)	Perimeter	CFIT	0.19
Rushton (1997)	100 East Asian-American 7-year-olds, 54% female	Perimeter	WISC	0.21
Tramo et al. (1998)	20 individuals (10 pairs of identical twins) aged 24–43 years	Perimeter	WAIS-R	0.14
Tan et al. (1999)	54 female university students in Turkey	Capacity	CFIT	0.55
Tan et al. (1999)	49 male university students in Turkey	Capacity	CFIT	0.29
Ivanovic, Olivares, Castro, and Ivanovic (1996).	4,124 school children of both sexes aged 6–17 years in Chile	Perimeter-for-age	School grades	0.24
Ivanovic, Forno, Castro, and Ivanovic (2000)	4,509 5- to 22-year-old male and female students in Chile	Perimeter-for-age	Raven's Matrices	0.22
Wickett et al. (2000)	68 individuals (34 pairs of brothers) aged 20–35 years	Perimeter	*g*, from MAB and other tests	0.19
Ivanovic et al. (2004)	47 male 18-year-old high school students in Chile selected from the richest and poorest counties	Perimeter-for-age	WAIS-R	0.50
Ivanovic et al. (2004)	49 female 18-year-old high school students in Chile selected from the richest and poorest counties	Perimeter-for-age	WAIS-R	0.40
Beckett et al. (2006)	77 Romanian children (66% male) adopted into UK families with head size measured before age 43 months and GMA scores measured at 11 years	Perimeter	4 scales from WISC-III (UK)	0.27
Bates (2007)	164 university students aged 21 years (124 female, 40 male)	Perimeter	Raven's Matrices	0.21
Rushton, Cvorovic, et al. (2007)	323 16- to 66-year-old Serbian Roma Gypsies (111 males, 212 females)	Capacity	Raven's Matrices	0.13
Rushton, Vernon, et al. (2007)	239 18- to 25-year-old Canadians (108 males, 131 females; 77% White)	Perimeter	*g*, from MAB and Wonderlic	0.19
Number of samples: 59				
Total *N*: 63,405				
Unweighted mean *r*: 0.21				
*N*-weighted mean *r*: 0.20				

*Note*. CFIT, Culture-Free Intelligence Test; MAB, Multidimensional Aptitude Battery; NART, New Adult Reading Test; PMAT, Primary Mental Abilities Test; WAIS-R, Wechsler Adult Intelligence Scale-Revised; WISC, Wechsler Intelligence Scale for Children; WISC-III (UK), Wechsler Intelligence Scale for Children, 3rd Edition, UK standardization; Wonderlic, Wonderlic Personnel Test.
